# Supplementary material for: Fungal-Modified Lignin-Enhanced Physicochemical Properties of Collagen-Based Composite Films
Source: J Fungi (Basel). 2022 Dec 16;8(12):1303. doi: 10.3390/jof8121303 (PMC9783068; doi:10.3390/jof8121303)
Supplement: Supplementary file 1 [file jof-08-01303-s001.zip › jof-2078988-SI.pdf]

Fungal modified lignin enhanced physicochemical properties of collagen-based composite films

Alitenai Tunuhe[1†], Pengyang Liu[1†], Mati Ullah[1], Su Sun[1,2], Hua Xie[3], Fuying Ma[1], Hongbo Yu[1], Yaxian Zhou[3*], Shangxian Xie[1*]

[1] Department of Biotechnology, Key Laboratory of Molecular Biophysics of MOE, College of Life Science and Technology, Huazhong University of Science and Technolog, Wuhan 430074, China

[2] College of Urban Construction, Wuchang Shouyi University, Wuhan 430074, China

[3] Guangxi Shenguan Collagen Technology Research Institute, Guangxi Shenguan Collagen Biological Group, Wuzhou 543000, China

* Correspondence: shangxian_xie@hust.edu.cn (S. X.); xiasheng @vip.163.com (Y. Z.); Tel.: +86-27-87792108 (S. X.); +86-0774-2035538(Y. Z.)

† These authors contributed equally to this work.

List of Supplementary Material

Table S1. The relative abundance of aromatic compounds by unmodified and biomodification APL as determined by GC-MS

Table S2. Typical chemical shifts and integration regions for APL in a 31P NMR spectrum

Table S3. Hydroxyl group contents of APL as determined by 31P NMR analysis

Figure S1. Quantitative 31P NMR spectrum of APL using cyclohexanol as internal standard

Table S1. The relative abundance of aromatic compounds by unmodified and biomodification APL as determined by GC-MS

	Aromatic compounds	AC	AF	AB	AE	AD	AX
1	Guaiacol	0.0945	0.0766	0.0532	0.0753	0.0174	0.0478
2	Benzoic Acid	0.0554	0.0477	0.0898	0.0430	0.0308	-
3	Benzeneacetic acid	0.0450	0.0501	-	0.0362	-	-
4	4-Hydroxybenzaldehyde	0.5529	0.4126	0.3892	0.5037	0.0555	-
5	4'-Hydroxyacetophenone	-	0.1830	0.3011	0.1399	-	0.1533
6	4-Isopropylphenol	0.2182	-	-	-	-	-
7	2,4-Dihydroxybenzaldehyde	-	-	-	0.1062	-	-
8	Butylated Hydroxytoluene	0.1796	0.1540	0.1643	0.1420	0.0879	0.1397
9	Vanillin	0.1676	0.2404	0.2658	0.3185	0.0319	0.0579
10	3-Hydroxybenzoic acid	0.0243	0.0253	0.0319	0.0235	0.0104	-
11	Acetovanillone	-	-	0.1833	0.1750	-	-
12	4-Hydroxybenzoic acid	0.3795	0.3421	0.5875	0.3121	0.5797	0.0679
13	4-Hydroxybenzeneacetic acid	0.0828	0.0790	0.1274	0.0658	0.0327	0.1020
14	Phloretic acid	-	-	0.1232	0.0973	0.0461	-
15	Vanillic Acid	-	-	0.2977	-	0.1999	-
16	Acetosyringon	0.8145	-	-	-	-	-
17	Homovanillic Acid	0.1353	0.1021	0.1259	-	0.0435	0.1188
18	4-Coumaric acid	9.0262	8.2180	7.1692	7.1723	2.4794	1.1333
19	Vanillylmandelic acid	-	-	0.1465	-	0.0189	0.0763
20	Syringic acid	0.5774	0.4618	0.1939	0.3196	0.1108	0.0891
21	(Z)-2-hydroxy-3-(4-hydroxyphenyl) acrylate	-	-	0.1288	0.1658	-	-
22	Ferulic acid	2.0813	1.3407	0.4910	1.3120	0.4039	0.3279
23	2-hydroxy-3-(4-hydroxy-3-methoxyphenyl) propanoic acid	0.0853	0.1283	0.1096	0.0645	0.0222	0.0424
24	m-Coumaric acid	-	0.0959	-	-	-	0.0868
	**Total**	**14.5198**	**11.9573**	**10.9793**	**11.0727**	**4.1708**	**2.4431**

Table S2. Typical chemical shifts and integration regions for APL in a 31P NMR spectrum

structure	δ(ppm)
(1) Aliphatic OH	145.4-150.0
(1) Phenols	133.6-144.0
C5 substituted	140.0-144.5
Syringyl	~142.7
Guaiacyl	139.0-140.2
Catechol	~138.9

Table S3. Hydroxyl group contents of APL as determined by 31P NMR analysis

	Aliphatic OH mmol g^{-1}	Phenolic, mmol g^{-1}					COOH mmol g^{-1}
		C5 substituted	Syringyl	Guaiacyl	Catechol	Phenols	
AC	1.477	0	0	0	0	0	0
AF	1.116	0.014	0.018	0.022	0	0.162	0
AE	1.242	0	0	0.022	0.007	0.007	0
AB	1.101	0.069	0.036	0.029	0.014	0.119	0.022
AD	1.217	0.181	0.018	0.047	0.014	0.134	0.087
AX	0.772	0	0	0.003	0	0	0.054

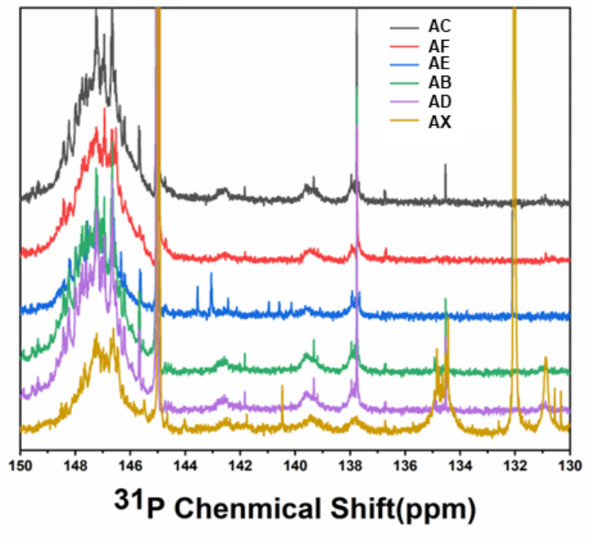

Figure S1. Quantitative 31P NMR spectrum of APL using cyclohexanol as internal standard
